# Development of a breast cancer screening protocol to use automated breast ultrasound in a local setting

**DOI:** 10.3389/fpubh.2022.1071317

**Published:** 2023-01-04

**Authors:** Judit Tittmann, Marcell Csanádi, Tamás Ágh, György Széles, Zoltán Vokó, Árpád Kallai

**Affiliations:** ^1^Semmelweis University, Center for Health Technology Assessment, Budapest, Hungary; ^2^Syreon Research Institute, Budapest, Hungary; ^3^Csongrád-Csanád Regional Health Center, Hódmezovásárhely, Hungary

**Keywords:** mammography, screening protocol, quality indicators, stakeholder engagement, breast cancer screening, automated breast ultrasound

## Abstract

**Introduction:**

The sensitivity of mammography screening is lower in women with dense breast. Increasing the efficacy of breast cancer screening have received special attention recently. The automated breast ultrasound (ABUS) shows promising results to complement mammography. Our aim was to expand the existing breast cancer screening protocol with ABUS within a Hungarian pilot project.

**Methods:**

First, we developed a protocol for the screening process focusing on integrating ABUS to the current practice. Consensus among clinical experts was achieved considering information from the literature and the actual opportunities of the hospital. Then we developed a protocol for evaluation that ensures systematic data collection and monitoring of screening with mammography and ABUS. We identified indicators based on international standards and adapted them to local setting. We considered their feasibility from the data source and timeframe perspective. The protocol was developed in a partnership of researchers, clinicians and hospital managers.

**Results:**

The process of screening activity was described in a detailed flowchart. Human and technological resource requirements and communication activities were defined. We listed 23 monitoring indicators to evaluate the screening program and checked the feasibility to calculate these indicators based on local data collection and other sources. Partnership between researchers experienced in planning and evaluating screening programs, interested clinicians, and hospital managers resulted in a locally implementable, evidence-based screening protocol.

**Discussion:**

The experience and knowledge gained on the implementation of the ABUS technology could generate real-world data to support the decision on using the technology at national level.

## Introduction

The importance of early detection and the need for breast cancer screening are well-recognized. The Council of the European Union (EU) issued recommendations in 2003 that called on all EU countries to take common action to implement national, population-based screening programs for 3 cancer sites (i.e., breast, cervical and colorectal cancers). Mammography is currently used for breast cancer screening in all population-based European programs and digital mammography has completely replaced film-screen mammography in the great majority of the countries (1). There is substantial evidence that organized screening with mammography reduces breast cancer mortality in the target population. However, this impact shows large differences, which reflects more on how screening is implemented, rather than the effectiveness of screening in general (2).

On the other hand, the sensitivity of mammography in some patient subgroups is significantly lower than in the full target population (3). Therefore, its impact cannot be generalized and individual characteristics should be considered. One of these parameters is breast density, which is the ratio of breast adipose tissue to fibroglandular elements. This ratio strongly influences the ability of screening mammography to detect lesions, the denser the breast, the larger the probability that the lesion may be obscured. Generally, breast density is classified into one of the four Breast Imaging Reporting & Data System (BI-RADS) categories. About 35% of women undergoing mammography have dense (BI-RADS C) and about 10% have extreme dense breast (BI-RADS D) (4). While the sensitivity of mammography is about 90% among women with fatty-breast (BI-RADS A), only ~65–75% among those with BI-RADS D (5). This is because the tissue elements that give the density of the breast, appear in the mammography image as radiation absorber white, as the cancerous lumps to be detected as well. Thus, lesions in the breast are more likely to remain unnoticed. This is called “masking effect.”

Increased breast density is assumed to be an independent risk factor of breast cancer. Women with dense or extremely dense breast are more likely to be diagnosed with breast cancer in their lifetime than women from BI-RADS category B (6). The association between breast density and the risk of breast cancer is confirmed by several studies (7), but the background mechanism and the degree of this effect are yet to be clarified.

As these issues are well-acknowledged, increasing the efficacy of breast cancer screening programs have received special attention in recent years and there have been multiple technological developments for such purpose. Most of these were tested as a complementary tool to the mammography examination. One of these methods is automated breast ultrasound (ABUS).

Ultrasound has long been used to examine the breast, because it provides a good overview of the tissue, it is relatively inexpensive, it does not use ionizing radiation and it is well-tolerated by patients (8). However, the examination is time consuming and the test result is strongly influenced by the experience of the operator. Another important disadvantage is that the records are not stored, thus subsequent review and assessment of the images is not possible (9). ABUS has been developed to overcome these limitations. This technology allows to separate the process of acquisition and interpretation. Acquisition is made by the device automatically. Hundreds of 2D images of the breast are taken from anteroposterior, lateral, medial views (referring to the position of the transducer pod against the breast during the scanning process) as the scan box moves in a cranio-caudal direction (10). The whole process takes about 15–20 min (11). The data are saved and transferred to a dedicated workstation. The 3D multiplanar reconstruction is automatically performed by the dedicated software, which enables a comprehensive analysis of the breast tissue.

ABUS provides the radiologist a thorough, detailed review of the breast structure. Thus, the tumor masking effect of the dense breast can be considerably reduced. In case the mammographic examination was supplemented with ABUS, the tumor detection rate has improved (RR = 1.44; 95% CI, 1.16–1.78) (12). Tumors detected only with this technology are tend to be smaller in size and are in an earlier stage, which promises greater success in the cancer treatment (13). However, with ABUS, an acceptable increase in the number of patients, recalled after the screening examination could be observed (14). It is important to add that the recall rate is influenced by several factors (e.g., the method of evaluating the recordings, the algorithm for the recalls, the learning curve of the reader), by which the number of patient recalled can be further decreased (15).

The objective of this study was to expand the existing breast cancer screening protocol with ABUS within the framework of a local pilot program in Hungary in a city hospital. This could create the opportunity to gain experience and knowledge on implementation of the technology and can generate real-world data to support the decision on using this technology at national level.

## Materials and methods

The protocol was developed in a partnership of researchers experienced in planning and evaluating screening programs and local stakeholders including clinicians and hospital managers. The pilot program to complement mammography screening with ABUS is implemented at the regional mammography center of the Csongrád Megyei Egészségügyi Ellátó Központ Hódmezővásárhely-Makó, Hódmezővásárhely, Hungary. The protocol development was conducted in two steps. First, a protocol was developed for the screening process focusing on integrating ABUS to the current screening practice. This was followed by the development of a protocol for evaluation that ensures the systematic data collection, the monitoring and the comprehensive assessment of the cancer screening with the addition of the ABUS.

### Protocol for the screening process

To create a clear and comprehensive structure for the protocol, international guidelines and recommendations on the implementation of cancer screening were reviewed and the World Health Organization (WHO) handbook for guideline development was considered (16). Based on these documents, we determined the main chapters of the protocol: scope of the protocol, target population for screening, technical parameters, process of screening activity with a flow chart, human capacities for screening, communication about screening, requirements to implement the protocol. Subsections for each chapter (i.e., medical, non-medical staff for human capacities or physical, educational and financial requirements of screening) were formulated.

To define the content of these chapters, multiple sources were used. We primarily considered international guidelines and recommendations on breast cancer screening. The latest Hungarian national protocol on breast cancer and early diagnosis was published in 2008. Although, it became outdated in 2010, it was an important cornerstone, since it contains basic considerations for the process of screening with mammography (17). In addition, a Hungarian consensus paper on the methods for breast cancer detection provided some information about ABUS as well, which was interpreted from the perspective of the protocol (18). Finally, a recently published comprehensive review about ABUS was also considered (19).

After the review of these materials, the screening protocol was developed through an iterative process of discussions with the clinicians and the management of the hospital. We aimed to create consensus among the stakeholders taking into account the information from the literature and the actual context in the hospital. This process was supported by a predefined list of questions focusing on proposing feasible solutions for the hospital. The questionnaire in Supplementary Table S1 formed the basis of online discussions.

### Protocol for evaluation

To ensure the evaluation of ABUS for screening, another part of the protocol was developed for data collection. The starting point was the list indicators and their definitions developed by EU-TOPIA H2020 project, that aimed to perform a comprehensive analysis and harmonization of breast, colorectal and cervical cancer screening programs in the EU (20). Based on this work, the main categories of the indicators, the indicators themselves and their calculation methods were defined. Further characteristics of the indicators were also defined regarding their feasibility during the planned pilot program (~6–9 months) taking into account the potential data sources. The final form of the evaluation protocol was created after comprehensive online discussions with clinical experts.

The study protocol for evaluation was approved by the Regional and Institutional Committee of Medical Science and Research Ethics at Szeged University (registration number: 771-462/2022). Data collection to evaluate breast screening with mammography and ABUS has begun in the framework of the pilot program on April 15, 2022, at the regional mammography center in Hódmezővásárhely.

## Results

### Protocol for the screening process

The scope of the protocol includes the application of ABUS as a supplemental imaging method for mammography within a pilot study in a regional mammography center. The target population of ABUS examination was defined as those asymptomatic women, who participated in the periodic mammography screening (i.e., women aged 45–65 years in every 2 year), their mammogram was negative and showed high or extreme high breast density.

**Figure 1:**
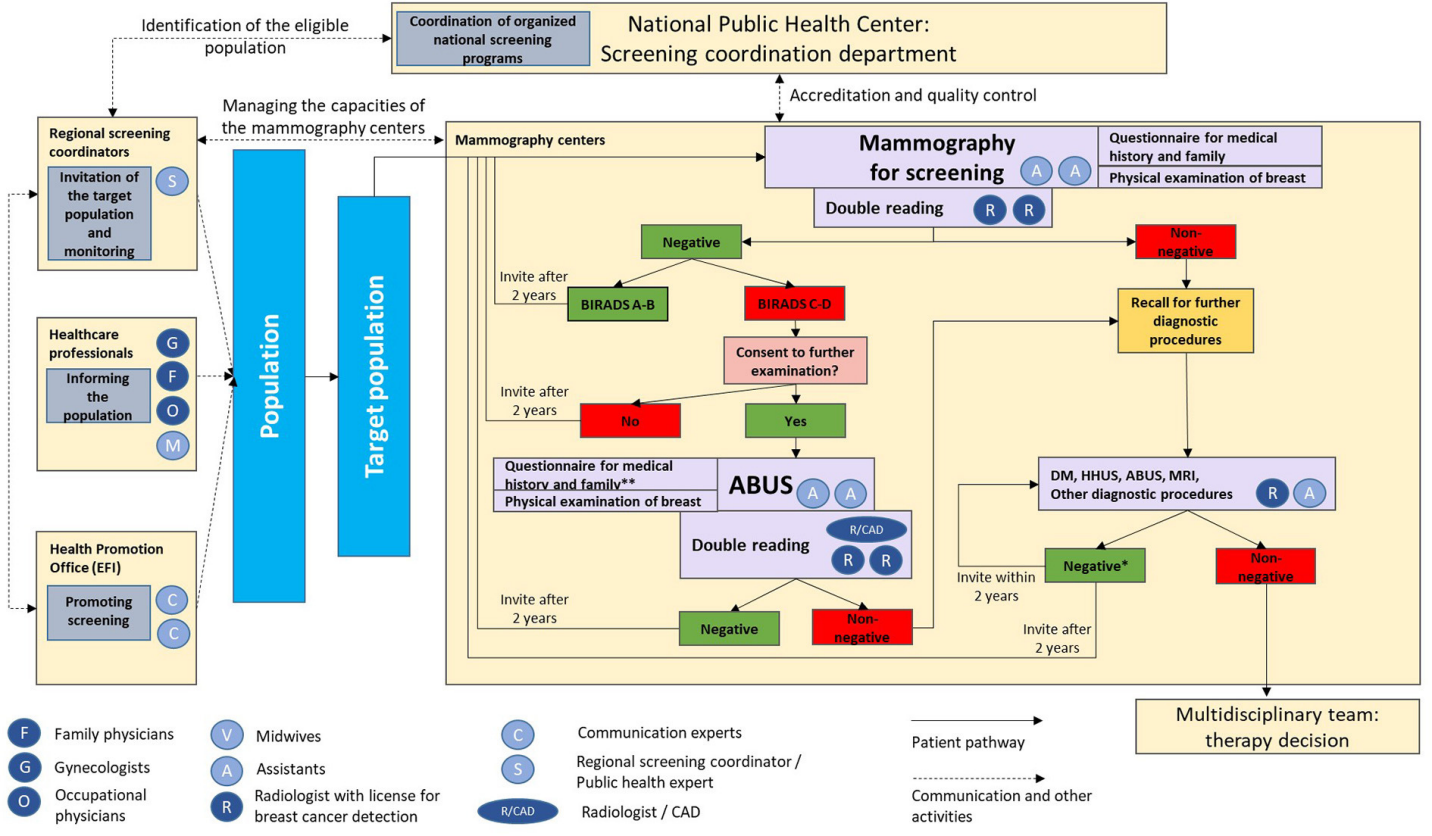
Process of the screening activity with the incorporation of automated breast ultrasound. ABUS, automated breast ultrasound; BIRADS, Breast Imaging Reporting & Data System; CAD, computer-aided artificial intelligence detection; DM, digital mammography; HHUS, hand-held ultrasound; MRI, magnetic resonance imaging. *Based on the result of the first non-negative mammography; **in case of missing or outdated information.

The process of the screening activity is shown in a detailed flow chart (Figure 1). For the organized, nationwide mammography screening, women are invited personally *via* letters from the regional screening coordinator. Those who are attending, fill in a paper-based short questionnaire about the risk of breast cancer based on personal and family history. Thereafter, a physical examination of the breast is performed by the assistant, which is still part of the traditional screening process in Hungary. Then the mammography is performed. After evaluation of the mammogram, ABUS examination is offered to women with dense or extreme dense breasts by the staff of the mammography center. Without further referrals, women can attend the ABUS examination.

ABUS acquisition is accomplished by a qualified assistant. After preparing and positioning the patient, the device performs the recording automatically. The data are exported to a workstation, where they are processed and reconstructed to allow 3D visualization of the breast. As in the case of mammography, double reading of the records is recommended. In case computer-aided artificial intelligence detection (CAD) system is available, one radiologist and the use of CAD system can fulfill the requirement of double-reading. If CAD system is not accessible, the evaluation of the images is made by two radiologists independently. Currently the latter option is implemented at the hospital for which this protocol is prepared. Differences in interpretation are reconciled by consensus between the two physicians. Any perceived lesions are documented according to the international standards used in the hospital. If lesion were detected by the ABUS, women are informed about the need of further diagnostic procedures.

In the case of negative result, women undergo the periodic mammographic screening after 2 years, in accordance with the national breast cancer screening protocol. The current protocol considers ABUS even after the subsequent mammography examinations for woman with dense breast.

The addition of ABUS examination does not impose a significant additional burden on the center in terms of human resources. For administrative tasks, one person is required. It is recommended to provide two people to perform the clinical assistant duties. In order to carry out a double reading of the records, it is essential to guarantee two radiologists as the CAD system is not yet available. From the technical point of view, in addition to the ABUS machine, the center must also provide an appropriate information technology (IT) background, which allows the recordings to be stored, processed and displayed. From the financial perspective, there is no reimbursement currently for the examination; therefore, the hospital must cover the costs from the total budget that is received for screening related activities.

The communication strategy related to breast cancer is an important element of the protocol as it strongly influences the success of the program. The National Public Health Center, which is responsible for organizing mammographic screening, the health professionals performing mammographic screening and the primary care workers are involved in the development of society's awareness about breast cancer screening and the importance of breast density. Women recommended for ABUS based on their mammogram results are approached through the communication channel preferred by the patient (e.g., phone or e-mail). They are informed about the results of the mammogram and the details of the ABUS examination during a personal consultation. Discussions to shared decision-making with women require up-to-date knowledge of physicians on the available imaging techniques in breast cancer. Moreover, education and training programs for specialists conducting ABUS examination are fundamental cornerstones of the successful screening program, which were considered in the protocol. Both the producers and professional associations provide technical and medical trainings and workshops for radiologist and medical personals, as well.

### Protocol for evaluation

We defined 23 indicators for the comprehensive evaluation, which were grouped into four major categories: program indicators (*n* = 13), test indicators (*n* = 5), cost indicators (*n* = 2), long-term clinical indicators (*n* = 3). For these indicators a detailed description was provided in Supplementary Table S2.

There were 13 indicators, which were considered relevant on a short-term basis (i.e., within a year) and these require regular data processing even in a short time-window. One indicator was considered relevant on a short and long term as well, while in case of 9 indicators long term data collection would be required for the calculation. Accordingly, 14 indicators were considered possible to calculate within the scope of the pilot study. The calculation of 6 indicators is conditional based on the feasibility of additional data extraction from external sources, while 3 indicators will most likely to be calculated only on the long term, after the pilot project (cancer-specific survival, cause-specific mortality, breast cancer incidence).

Those indicators, which were considered possible to collect, the primary data source is the database of the mammography center, which should be manually processed due to the lack of automated data collection practice. For many indicators, the medical IT system of the hospital is also a primary source. The most important limitation of the data collection is that the information about breast density is currently not possible to collect automatically since it is not recorded in the medical IT system of the hospital. Therefore, manual data collection is required.

## Discussion

Research on the novel screening or diagnostic technologies are rapidly progressing and many innovative technologies are under development for detecting cancer at an early stage. Thus, regular monitoring of the organizational and structural framework of screening programs is necessary to achieve their optimal performance and to take advantage of new advances. Possible directions for further development of screening programs could be (1) to improve the efficiency of organizing screening activities, (2) to better target the population for screening (e.g., *via* personalized screening on the basis of individual risk factors), (3) to implement targeted screening programs for certain social strata (e.g., lower socio-economic status), (4) to improve the monitoring of screening programs, and (5) to incorporate technological developments into existing screening protocols (21–23). In the present report we specifically focused on the latest opportunity.

Even though breast density has been recognized as an important factor that significantly reduces the sensitivity of mammography, currently there is still a consensus to consider mammography as the gold standard imaging method for breast cancer screening (24). However, we are currently on the verge of change. The current EU Council Recommendation on breast cancer screening is almost 20 years old. According to the research results collected over the years on effective cancer screening, the latest proposal for update of the EU Council Recommendation proposes to extend the age of the target population to 45–74 years and to consider specific imaging screening methods for women with particularly dense breasts (25). Furthermore, another EU report published in 2022 as well, provided a recommendation on using supplemental magnetic resonance imaging (MRI) screening to improve the sensitivity of breast screening in women with dense breasts (26). However, the feasibility of implementation should also be taken into account in terms of the available infrastructure, equity in access for the entire target population, the acceptance of the examination by the target population and the rational use of healthcare resources. According to EUSOBI recommendation, when the MRI examination is not available, a mammography supplemented with ultrasound examination is suggested for women with extremely dense breasts (3).

In this paper we described a protocol development with ABUS, which was based on the considerations and the engagement of different stakeholders. We successfully designed a screening protocol and started its implementation in a pilot program targeting an underdiagnosed subpopulation by considering the current literature on ABUS and achieving consensus among researchers, clinicians, and managers. As data will be obtained under real-word conditions we hope to contribute to the evidence base of breast cancer screening, which can be utilized in a potential roll-out. In order to evaluate the feasibility of implementing a new screening modality, it is essential to examine its economic consequences as well. A recent budget impact analysis about ABUS, conducted in Italy, found an increase in the screening phase expenditure, but economic advantage related to subsequent treatment of diagnosed patients (27). The program and the developed protocol for evaluation also provides an opportunity to examine the economic aspects of ABUS by taking local conditions and environment into account. Initiating a pilot program and using its experience and data is a frequently applied approach in the field of cancer screening. This approach was followed in Hungary as well for instance in case of breast or colorectal screening earlier (28, 29). However, this requires not only the comprehensive evaluation of the pilot project but also a wider consultation with relevant national-level stakeholders in case of promising results.

It is our hope that the implication of our work also goes beyond the ABUS technology as the method of the protocol development is an example of a stakeholder engagement-based project planning and implementation. The co-creation of the screening protocol by researchers and local stakeholders ensured that it is based on sound scientific evidence and takes into account the local context. In addition, our protocol for evaluation was developed by considering international standards, therefore, it could be used as the basis for establishing a comprehensive monitoring system of the national screening program. This reflects on a need, that has never been fully addressed before in Hungary despite the over two decades of breast cancer screening history. Unfortunately, those complex and long-term indicators, which are required for the monitoring and comprehensive evaluation of a screening program (30), has never been published mainly due to the lack of systematic data collection.

## Data availability statement

The raw data supporting the conclusions of this article will be made available by the authors, without undue reservation.

## Ethics statement

The studies involving human participants were reviewed and approved by Regional and Institutional Committee of Medical Science and Research Ethics at Szeged University (Registration Number: 771-462/2022). The patients/participants provided their written informed consent to participate in this study.

## Author contributions

JT, MC, and ZV wrote the first draft of the manuscript. All authors contributed to conception and design of the screening protocol. All authors contributed to manuscript revision, read, and approved the submitted version.
