# Supplementary material for: Development of a breast cancer screening protocol to use automated breast ultrasound in a local setting
Source: Front Public Health. 2023 Jan 4;10:1071317. doi: 10.3389/fpubh.2022.1071317 (PMC9846565; doi:10.3389/fpubh.2022.1071317)
Supplement: Supplementary file 1 [file Data_Sheet_1.PDF]

Supplementary Table S1. Predefined list of questions focusing on proposing a feasible option for the hospital on implementing ABUS

Question #1: Who is the target audience of the protocol? Is it medical professional, the management of the hospital or others?
Question #2: Who is responsible for implementing the protocol?
Question #3: What risk factors should be considered when the target population for screening is defined?
Question #4: Which exclusion criteria should be taken into account besides the risk factors?
Question #5: How important it is to take into account the imaging parameters of the screening tool? If important, which parameters are the most important ones?
Question #6: How important it is to take into account the available softwares for evaluating the images? If important, which software is important for the screening activity?
Question #7: Who should be involved in the organization of the screening activity?
Question #8: What is the process of the screening? Who and how will record the screening parameters?
Question #9: How and when should the assessment of the imaging performed? What parameters are defined? Who will define these?
Question #10: What is the procedure to follow after the negative screening result?
Question #11: What is the procedure after the non-negative screening result? What diagnostic procedures should be done? Who is responsible for the diagnostic procedures?
Question #12: What is the procedure after suspected but non-malignant cases of the screening?
Question #13: What is the procedure after false-positive results?
Question #14: How many person are available to complete the required administrative work for the entire screening procedure?
Question #15: How many medical professionals are available for the screening procedure?
Question #16: How many non-medical professionals are available for the screening procedure?
Question #17: Is there human resource capacity for completing the other required work for to complete the screening procedure?
Question #18: Who is responsible for the communication towards the target population? How should the communication be done?
Question #19: How is the communication look like within the team responsible for screening?
Question #20: Who is responsible for the communication towards those who attended screening? How should the communication be done?
Question #21: What communication should be done to other fields (i.e. policymakers, medical professional societies)?
Question #22: What physical and material conditions are available to perform the screening?
Question #23: What professional requirements are met or missing to perform the screening? What educational activities were done and what activities are needed in the future?
Question #24: What are the financial considerations that are important for performing the screening?
Question #25: What are the requirements of the sustainability of the screening?
