# Supplementary material for: Development of a breast cancer screening protocol to use automated breast ultrasound in a local setting
Source: Front Public Health. 2023 Jan 4;10:1071317. doi: 10.3389/fpubh.2022.1071317 (PMC9846565; doi:10.3389/fpubh.2022.1071317)
Supplement: Supplementary file 2 [file Data_Sheet_2.PDF]

Supplementary Table S2. *List of indicators for the comprehensive evaluation of screening with ABUS*

Indicator name	Definition and calculation	Timeframe of data collection*
**Program indicators**		
Target population	N eligible for the screening based on the inclusion criteria	Short term
Coverage by invitation	N invited / N target population	Short term
Coverage by examination	N screened / N target population	Short term
Attendance	N screened / N invited	Short term
Test result	N with each test result / N screened	Short term
Referral after positive test results	N referred for further assessment / N screened	Short term
Compliance with further assessment	N attended in further assessment / N referred to further assessment	Short term
Biopsy rate	N with at least one biopsy / N screened	Short term
Proportion referred to treatment	N referred to cancer treatment / N attended in further assessment	Short term
Compliance with treatment	N started cancer treatment / N referred to cancer treatment	Short term
Detection rate	N with histological cancer diagnosis / N screened	Short term
Interval cancer rate	N with new cancer cases during screening interval / N screened	Long term
Attendance on subsequent screening	N screened in subsequent screening round / N invited for subsequent screening round	Long term
**Test indicators**		
Sensitivity of the screening test	N screen-detected carcinomas / N screen-detected carcinomas + N carcinomas not detected with screening (false-negative)	Long term
Specificity of the screening test	N true negative cases / N true negative cases + N false positive cases	Long term
Positive predictive value	N screen-detected carcinomas / N screen-detected carcinomas + N false positive cases	Short and long term
Negative predictive value	N true negative / N true negative + N carcinomas not detected with screening (false-negative)	Long term
False positive rate	N false positive / N false positive + N true negative	Long term
**Cost indicators**		
Cost of the invitation for the screening program	Costs associated with the communication tow the target population and other screening promotion activities (total cost or per capita cost)	Short term
Cost of the screening test and the analysis	Costs associated with the screening test and the evaluation of the tests (total cost or per capita cost)	Short term
**Long-term clinical indicators**		
Cancer-specific survival	N patients diagnosed with a specific cancer alive at the end of a specific time interval / N patient diagnosed with a specified cancer	Long term
Cause-specific mortality	N mortality related to cancer in a specified population / N study population	Long term
Incidence	N newly diagnosed patients with cancer / N study population	Long term

*Short term indicates that it could be collected and calculated within a year
